# Torque, chemistry and efficiency in molecular motors: a study of the rotary–chemical coupling in F_1_-ATPase

**DOI:** 10.1017/S0033583515000050

**Published:** 2015-11

**Authors:** Shayantani Mukherjee, Ram Prasad Bora, Arieh Warshel

**Affiliations:** Department of Chemistry, University of Southern California, 418 SGM Building, 3620 McClintock Avenue, Los Angeles, CA 90089-1062, USA

**Keywords:** rotary-chemical surface, F_0_F_1_-ATP synthase, bioenergetics, conformational surface

## Abstract

Detailed understanding of the action of biological molecular machines must overcome the challenge of gaining a clear knowledge of the corresponding free-energy landscape. An example for this is the elucidation of the nature of converting chemical energy to torque and work in the rotary molecular motor of F_1_-ATPase. A major part of the challenge involves understanding the rotary–chemical coupling from a non-phenomenological structure/energy description. Here we focused on using a coarse-grained model of F_1_-ATPase to generate a structure-based free-energy landscape of the rotary–chemical process of the whole system. In particular, we concentrated on exploring the possible impact of the position of the catalytic dwell on the efficiency and torque generation of the molecular machine. It was found that the experimentally observed torque can be reproduced with landscapes that have different positions for the catalytic dwell on the rotary–chemical surface. Thus, although the catalysis is undeniably required for torque generation, the experimentally observed position of the catalytic dwell at 80° might not have a clear advantage for the force generation by F_1_-ATPase. This further implies that the rotary–chemical couplings in these biological motors are quite robust and their efficiencies do not depend explicitly on the position of the catalytic dwells. Rather, the specific positioning of the dwells with respect to the rotational angle is a characteristic arising due to the structural construct of the molecular machine and might not bear any clear connection to the thermodynamic efficiency for the system.

## Introduction

Gaining a detailed understanding of the biological conversion of ATP to ADP is crucial for understanding the nature of energy transduction in life processes and also for practical understanding of the action of molecular motors (Boyer, 1997; Weber & Senior, 1997). At present it seems that some of the details of this process are not fully clear (Abrahams *et al*. 1994; Boyer, 1993, 1997; Fersht, 1999; Junge *et al*. 2009; Wang & Oster, 1998; Weber & Senior, 1997). One of the major unresolved questions is associated with the understanding of the way the free-energy of the chemical process is converted to the conformational changes that eventually lead to torque generation and work. This challenge became even more exciting in view of the remarkable progress made in the field of single molecule studies that directly visualized the unidirectional rotation of the central stalk (subunit γ) (Adachi *et al*. 2007; Okuno *et al*. 2008; Shimo-Kon *et al*. 2010). Among the many intriguing findings of the single molecule studies, one has been the discovery of the ATP binding and catalytic dwells (Adachi *et al*. 2007) where the γ subunit rotation stops intermittently during the ATP binding and catalytic phases occurring in the α/β subunits. Another recent discovery is that the rotational process continues in the right direction even when major parts of the γ subunit are removed (Chiwata *et al*. 2014; Uchihashi *et al*. 2011). These findings seem to present a problem for models that have postulated steric van der Waals (vdW) as the most important driving force behind the torque generation.

Our previous work started to explore the chemical reaction in F_1_-ATPase (Strajbl *et al*. 2003) and then proceeded to study the conformational landscape using a CG model (Mukherjee & Warshel, 2011), and generated the rotary–chemical surface elucidating the nature of the coupling in F_1_-ATPase. This work has reproduced the intriguing observation of the 80°/40° substep feature in F_1_-ATPase and has provided a clear understanding of the electrostatics driven process of the catalytic dwell around 80° as the central stalk γ rotates through its 120° steps (Mukherjee & Warshel, 2011). Another recent work (Mukherjee & Warshel, 2015) has provided a more quantitative understanding of the generated torque in the system under different conditions and elucidated the nature of the free-energy landscape required for the robust rotary–chemical coupling in F_1_-ATPase. Furthermore, this study dissected the role of the γ-subunit in details and demonstrated the importance of the electrostatics-based mechanism underlying the torque generation process in systems with truncated γ subunits (Mukherjee & Warshel, 2015). Although, our previous studies on F_1_-ATPase has elucidated the fundamental electrostatics-driven mechanism underlying the rotary–chemical coupling and torque generation processes, they have not asked whether the position of the catalytic dwell has a specific biological advantage for the efficiency of the system. Here we attempted to explore whether the position of the catalytic dwell plays a role in providing advantage to the overall torque generation and the utility of the chemical energy. It is found that the position of the dwell might not be essential for the efficiency of the torque generation in F_1_-ATPase.

## Outlining the problem

Despite the progress in elucidating the role of key structural elements of the F_1_-ATPase molecular motor, we still face several challenges of generating structure/energy correlations that will tell us how the motor really works. These challenges range from the need to gain clearer understanding of the reaction mechanism and the dependence of the reaction barrier on the conformational states, as well as the determination of the steps when the chemical energy is released and, of course, finding how this energy release is coupled to the conformational changes and to the overall torque generation process.

**Fig. 1:**
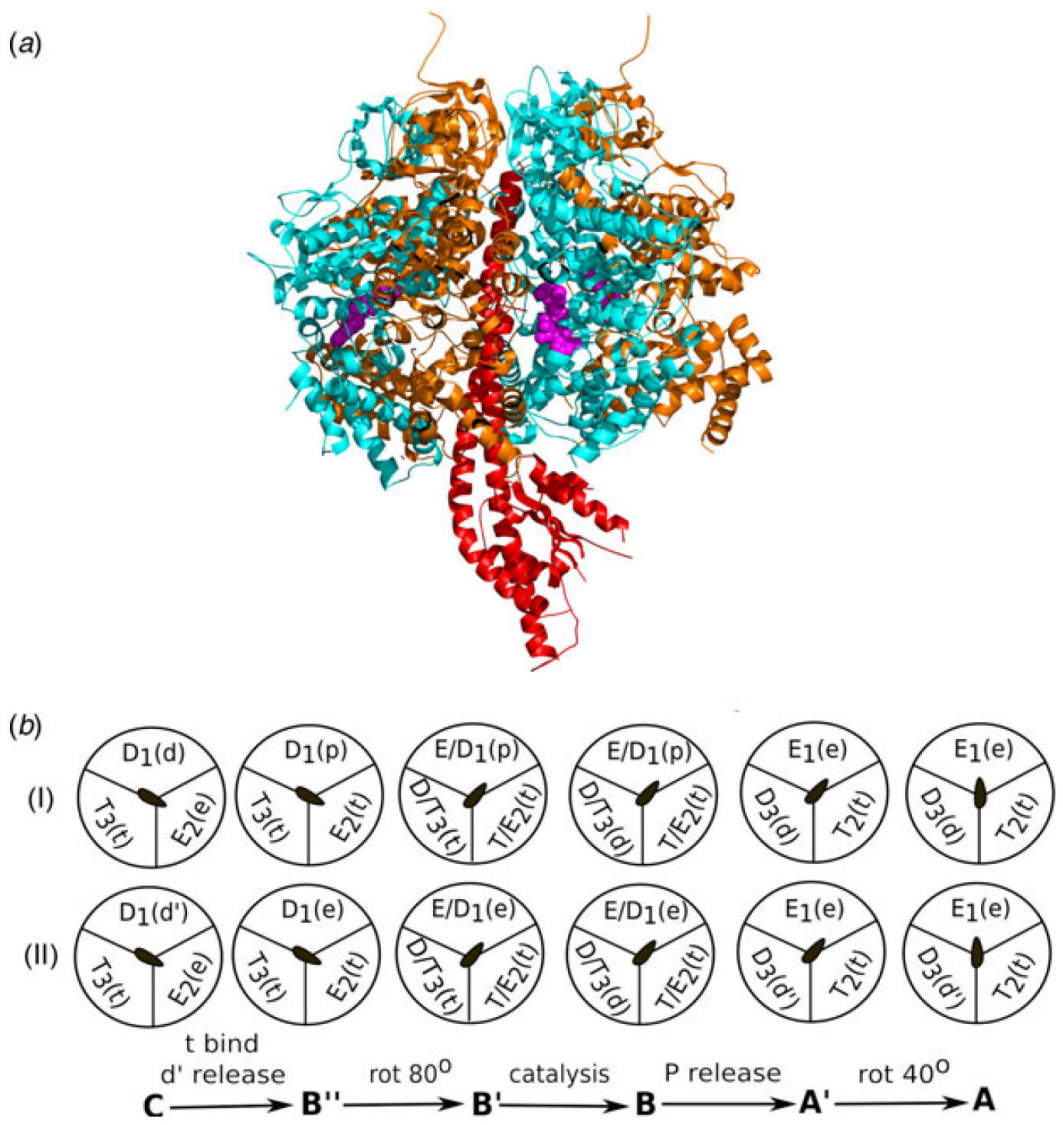
(*a*) Showing the atomistic structure of the F_1_-ATPase where the central stalk γ, subunits α and β are shown in red, orange and cyan, respectively, while bound nucleotides are in magenta. (*b*) Schematic representations of the ATP hydrolysis cycle during the 120° rotation of the γ subunit, where Scheme (I) and (II) describe two of the most likely mechanism. The stage between C to B may involve three nucleotides supporting tri-site mechanism (not shown explicitly) (Shimo-Kon *et al*. 2010).

The F_1_-ATPase subunit arrangements and the schematic ATPase cycle are presented in Fig. 1. The challenge is to reproduce the key observed facts from structure-based modeling approaches and then to use the corresponding computation to determine the origin of the different effects and their role in converting the chemical energy to work. Most of the theoretical studies that have attempted to resolve some of the above issues have not provided effective answers. An example is provided by Pu & Karplus (2008) that attempts to explore the substeps of the γ subunit rotation and to simulate the torque generated by this rotation. This work used a CG model and targeted molecular dynamics (TMD) to rotate the γ subunit between two observed structures. Unfortunately, the TMD was done in much smaller time scale than the experimentally observed millisecond time and more importantly, the simulations were done without considering the effect of the chemical energy on γ rotation. This means that the TMD actually probed the applied external force rather than the actual torque acting on the system due to the chemical process of ATP hydrolysis. The problems faced by the approach discussed above, as well as most other approaches that use large external force to explore the inherent molecular mechanisms of biological motors, partially reflect the fact that molecular understanding of the mechano-chemical processes requires to overcome the challenge of obtaining reliable free-energy landscapes for complex systems.

The above challenge has been addressed by our previous CG study (Mukherjee & Warshel, 2011), where we calculated the rotary–chemical free-energy landscape and obtained a reasonable rotation time, which is one of the key experimental observations about the action of the system. Furthermore, the rotary–chemical surface have provided an understanding of the reason for observing the catalytic dwell around 80° along the γ rotational path of F_1_-ATPase. In a subsequent work (Mukherjee & Warshel, 2015), we addressed the challenge of evaluating the actual torque obtained from the CG rotary–chemical surface (see the discussion below). However, our initial attempt to evaluate the torque has not considered the chemical barrier and only looked at the effect of releasing the chemical free energy at different possible stages of the conformational cycle.

**Fig. 2:**
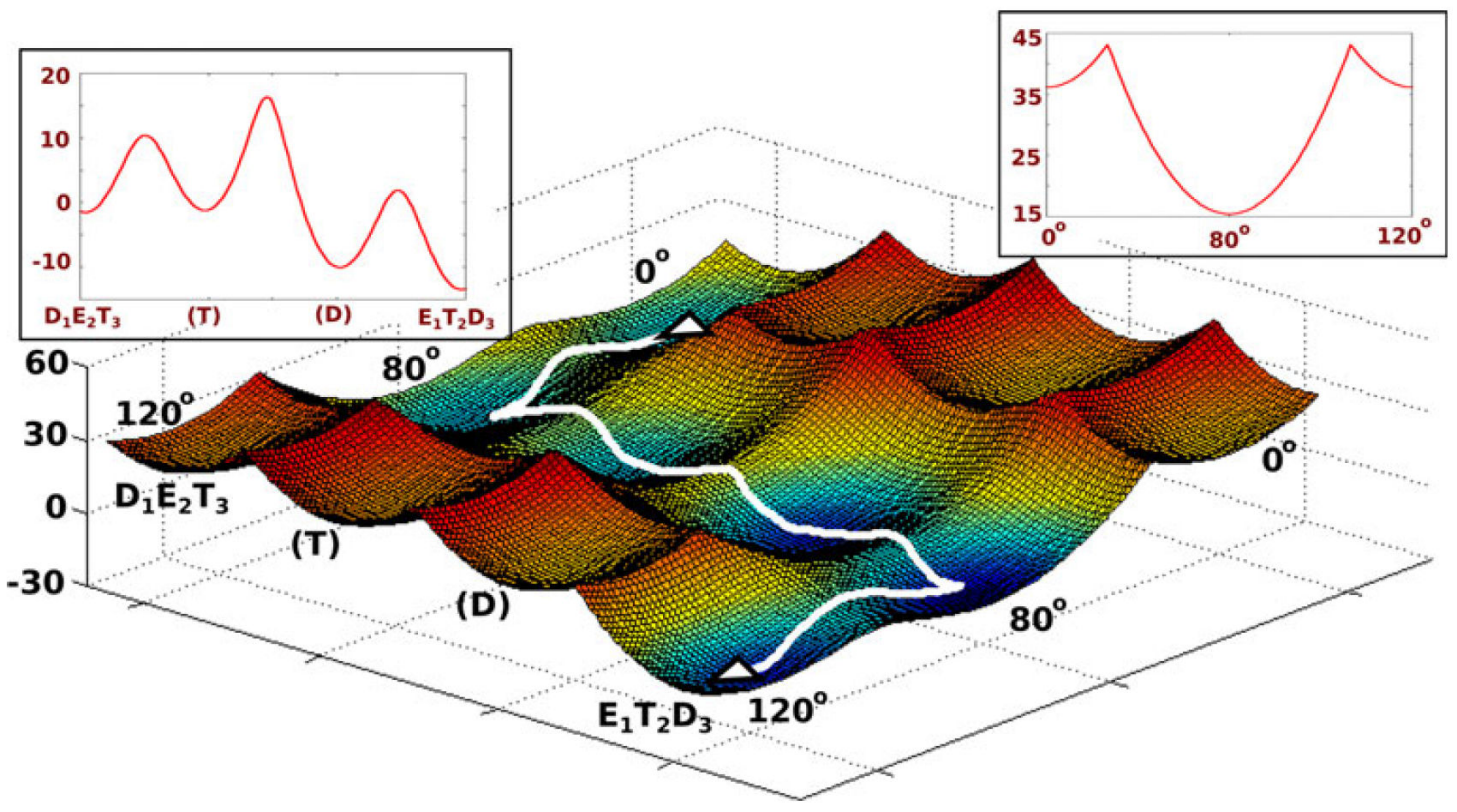
A schematic rotary–chemical surface for the ATP hydrolysis and the 120° γ rotation of F_1_-ATPase. The *X*-axis represents the change in the α/β catalytic subunits from D_1_E_2_T_3_ to E_1_T_2_D_3_, as well as the chemical transformation from ATP to ADP. The ATP and ADP states are designated by (T) and (D), respectively. The *Y* axis represents the γ rotation from 0° to 120°, while the *Z* axis represents the free energy of the system. The least free-energy path of the rotary–chemical process is shown with a white line on the 3D surface. The insets on the top left and top right corners show the free-energy profiles for γ = 80° and the chemical barriers at all values of γ, respectively.

Now, we would like to explore the relationship between the chemical free-energy landscape and the generated torque in a more rigorous way. Here we note the interesting experimental study of Watanabe *et al*. (2012), which explored the effect of mechanical modulation of the catalytic power of F_1_-ATPase. This study indicated that the barrier is reduced upon increase in the rotational angle of the γ subunit. Thus moving from γ = 0° to the catalytic dwell point (γ = 80°) results in a decrease in the barrier (although, moving past the 80° is blocked in our landscape due to very high electrostatic free energy). The substep feature of the electrostatic free-energy surface obtained in (Mukherjee & Warshel, 2011, 2015) is used to construct the schematic combined rotary–chemical surface shown in Fig. 2, where it is assumed that the chemical barrier has its lowest value at the point of the catalytic dwell (γ = 80°). This simplified surface and its modified versions can be used to explore whether there is any evolutionary advantage of having the lowest chemical barrier at a specific position along the γ rotational coordinate.

## Methods

In order to elucidate the molecular origin of the rotary–chemical coupling, it is important to model diverse events occurring at different spatiotemporal regime while being able to capture the structure function relationship at the same time. The theoretical modeling approach should provide a quantitative understanding of the dependence of the chemical barrier on the conformational changes, and the coupling between the conformational changes of the α/β and γ subunits. It is crucial to realize that the theoretically computed torque and the rotational time must be related to the underlying molecular free-energy landscape of the whole system in order to gain a quantitative knowledge of the biological function. To advance in this direction, we need to calculate the chemical and conformational features of the overall functional surface. The dependence of the chemical surface on the α/β conformational path can be evaluated using the EVB method (Kamerlin & Warshel, 2011) as was done in our preliminary study (Strajbl *et al*. 2003). However, for the purpose of the present study it is sufficient to use experimental estimates of the chemical barrier and explore the effect of the position of the lowest barrier on the torque generation in a parametric way.

The conformational landscape has been evaluated by a CG model (Messer *et al*. 2010) that describes the main chains by an explicit model and represents the side chains by a simplified united atom model. The model involves a unique treatment of the electrostatic energy including a self-energy term that makes it more reliable than other current models. The details of our model are given elsewhere (Dryga *et al*. 2011; Messer *et al*. 2010; Vicatos *et al*. 2014). After combining the chemical and conformational surfaces into an effective rotary–chemical surface (as shown in Fig. 2), our next task is to obtain the torque from the generated landscape and this can be done in several ways, including those discussed in (Arai *et al*. 2014; Panke *et al*. 2001; Sakaki *et al*. 2005). The first is to consider the free energy released upon rotation of the γ subunit (assuming that it is all transferred to the magnetic beads used to probe the rotation). In this case we have:

(1) $$\Delta G_{\text{Torque}}=\int \tau d\phi =\int \overset{‒}{\tau }d\phi ,$$

where τ is the angular torque and $\overset{‒}{\tau }$ is the average angular torque.

The relevant free energy can be evaluated from the potential of mean force (PMF) along the rotation coordinate, (denoted by ϕ), using a free-energy perturbation-type expression:

(2) $$e^{-\beta \Delta G_{\varphi_{m}}\to \varphi_{m+1}}=\frac{\int e^{-\beta [U_{X,\varphi_{m+1}}-U_{X,\varphi_{m}}]}e^{-\beta [U_{X,\varphi_{m}}]}dX\delta (\varphi -\varphi_{m})d\varphi }{\int e^{-\beta [U_{X,\varphi_{m}}]}dX\delta (\varphi -\varphi_{m})d\varphi }$$

The overall PMF can be evaluated by Monte Carlo (MC) simulations over the surface, although this can also be done by using LD simulations along with a mapping potential (Kato & Warshel, 2005).

## Results and discussion

Our analysis of the rotary–chemical coupling starts with the evaluation of the dependence of the ATP hydrolysis activation barrier on the conformational pathway (α/β subunit changes and γ subunit rotation). This was done here in a simplified way, assigning lower barrier at the catalytic dwell position (γ = 80°), following the observation of (Watanabe *et al*. 2012). However, to check the importance of the dwell position for torque generation and thermodynamic efficiency in the F_1_-ATPase system, we also considered different options of coupling the chemistry at other values of the rotational coordinate. The conformational landscape has already been determined in our previous works (Mukherjee & Warshel, 2011, 2015) and here we explored the effect of combining the conformational landscape with the chemical landscape at different position of the rotational coordinate.

**Fig. 3:**
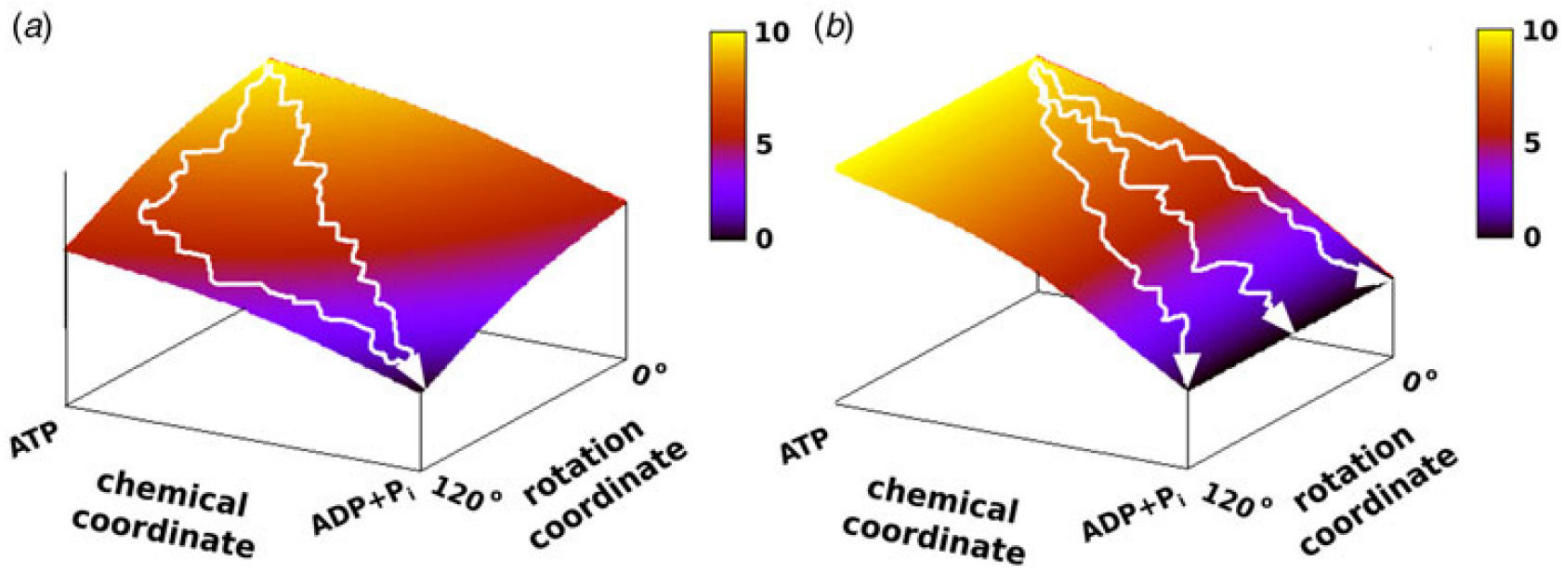
The relationship between the shape of the landscape and the generated torque is illustrated in the schematic landscapes for chemical and rotational coordinate (omitting the chemical or conformational barrier). Two extreme situations occur when the chemical free energy is tightly coupled (*a*) and loosely coupled (*b*) to the rotational coordinate. In (*a*) the chemical free energy decreases more when the rotational coordinate ϕ approaches 120° than when it approaches 0°. Hence the average trajectory ultimately move from ϕ = 0° to ϕ = 120° and the system generates torque. In (*b*) due to loose coupling of the rotational and chemical coordinate, the system can move to different rotational angles over time so that we obtain zero torque in the ϕ direction (this figure is a reproduction of Fig. 1 from (Mukherjee & Warshel, 2015).

Before moving to the actual calculations, it is useful to comment about the requirements underlying efficient torque generation in the F_1_-ATPase system. This point is illustrated by two schematic landscapes shown in Fig. 3, where Fig. 3*a* has a similar trend to the rotary–chemical coupling obtained in our previous studies that reveal a least energy path along the diagonal of the coupled surface (Mukherjee & Warshel, 2011, 2015). In the schematic landscape of Fig. 3*a*, the resultant torque that rotates the γ subunit reflects the fact that the lowest point on the coupled landscape occurs where the rotational and chemical states are at 120° and ADP +P_i_, respectively. In this case, the evolution of the system toward the 120° point hydrolyzes ATP and rotates the γ subunit in a unidirectional way. On the other hand, in Fig. 3*b* we have no net gradient for moving in the direction that rotates the γ from 0 to 120°. In this case, although the free energy decreases along the chemical coordinate as a result of the release of the chemical free energy, we still do not have an average net torque due to loss of effective coupling between the chemical and rotational coordinates. The relationship between the shapes of the landscape and the torque will guide us in the subsequent discussion and it will also serve to remind us that the torque cannot occur without proper coupling to the chemical energy.

**Fig. 4:**
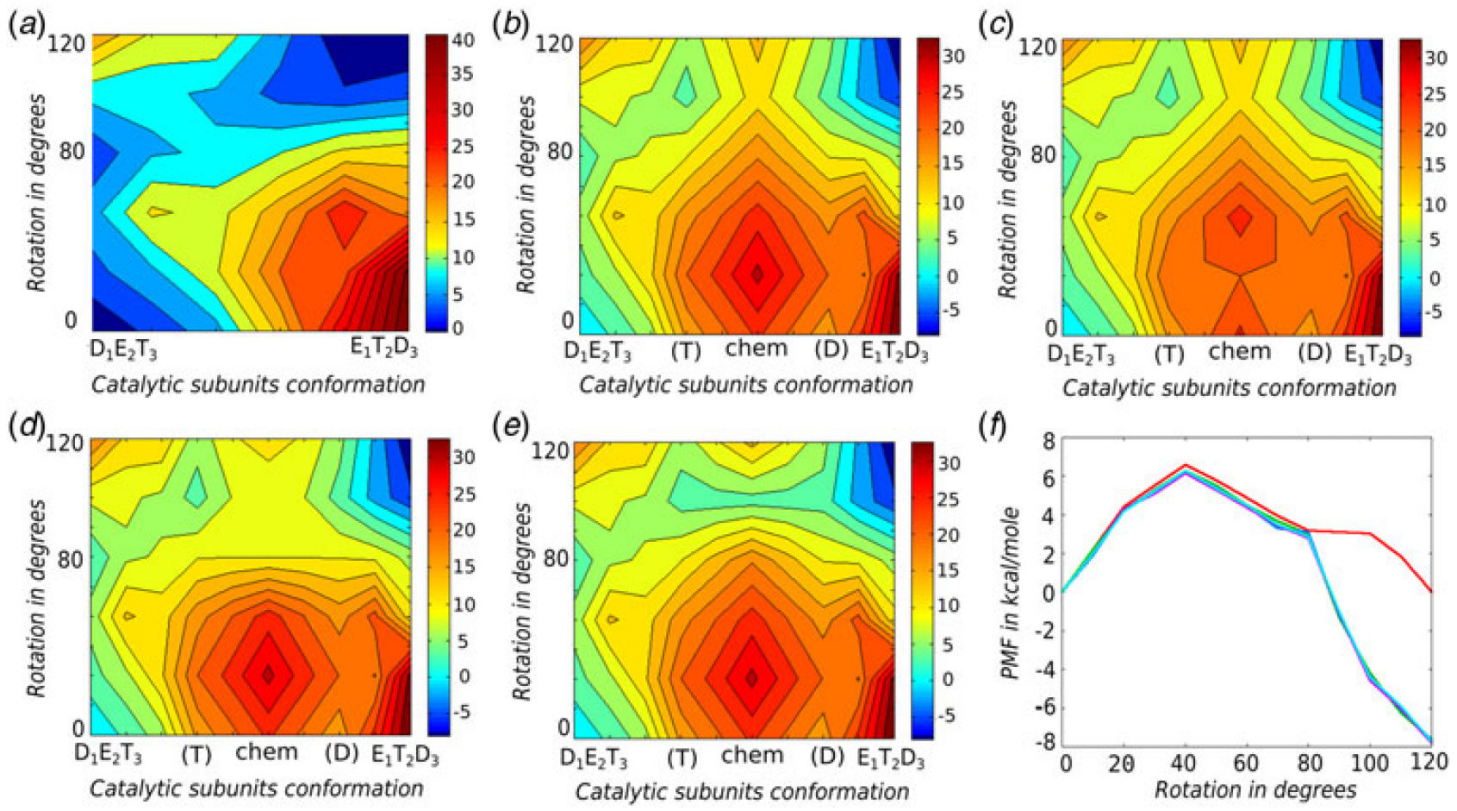
Illustrating the relationship between the chemical barriers at different γ positions with the generated torque. The surfaces represent a single 120° rotational event of F_1_-ATPase, where the *X*-axis represents the α/β conformational changes and the *Y*-axis represents the γ rotation. The coordinate for the chemical transformation (i.e. ATP to ADP) lies on the *X*-axis parallel to the catalytic subunit changes. The points (T) and (D) represent the ATP and ADP states of the chemical process, respectively, whereas (chem) represents the chemical barrier. The maps represent wild-type surfaces for the following cases: (*a*) without the chemical surface; (*b*) with similar chemical barrier for all rotational γ values; (*c*) with reduced chemical barrier at γ = 20°; (*d*) with reduced chemical barrier at γ = 80°; and (*e*) with reduced chemical barrier at γ = 100°. The PMF calculated from all surfaces are shown in (*f*) where the red, green, blue, magenta and cyan curves correspond to the surfaces of (*a*) to (*e*), respectively.

In order to study the advantage that F_1_-ATPase might have due to the catalytic dwell at γ = 80°, we added the chemical landscape at several regions of the conformational landscape and generated the surfaces shown in Fig. 4. In particular, we considered the case where chemical energy is not coupled to the conformational surface (Fig. 4*a*), where chemical barrier is similar throughout the rotational path (Fig. 4*b*), where the chemical barrier is lowest at γ = 20° (Fig. 4*c*), the experimentally observed case where the chemical barrier is lowest at γ = 80° (Fig. 4*d*) and where the chemical barrier is lowest at γ = 100° (Fig. 4*e*). With the different rotary–chemical surfaces at hand (Fig. 4*a*–*e*), we estimated the torque generated on each of those surfaces using the FEP formulation of Eq. (2). The corresponding PMFs are shown in Fig. 4*f*. As seen from the figure, there is negligible torque generation for the case when chemical energy is not coupled to the conformational landscape, which reflects the obvious fact that torque cannot be generated without expending chemical energy (red curve of Fig. 4*f*). On the other hand, significant torque is obtained for other cases where the chemical energy of ATP hydrolysis is coupled to the conformational surfaces. In the case of Fig. 4*d* we obtained a maximum free energy drop of around 7·6 kcal mol^−1^ between the 0° and 120° states in the direction of the rotational coordinate that can generate a maximum average torque of about 53 pN.nm on the γ rotational surface. This value lies close to the experimentally observed average torque range of 40–50 pN.nm (Noji *et al*. 1997; Panke *et al*. 2001). Now, the above torque was obtained by having the lowest chemical barrier at the observed catalytic dwell at γ = 80°. However, changing the position of the lowest chemical barrier with respect to the rotational angle (for e.g. γ = 20° or 100°) did not alter the generated torque significantly. Apparently, torque is also generated for the case where the chemical barrier is similar for all values of the rotational coordinate (Fig. 4*b*), implying that there is no specific advantage of having the catalytic dwell at a specific position, as long as the chemical energy is properly coupled to the rotational coordinate so that net unidirectional torque could be generated. Our finding indicates that the rotary–chemical surface is sufficiently robust so that the position of the dwell is not crucial for the overall efficiency of F_1_-ATPase, especially in terms of torque generation. This result is consistent with the observation (Furuike *et al*. 2011) that the bacterial V-type ATPase shows different rotational stepping behavior as compared to the F_1_-ATPase, while still functioning efficiently.

## Concluding remarks

In exploring the origin of the action of F_1_-ATPase and related systems, it is crucial to provide a clear structure function relationship of the rotary–chemical coupling. Such a relationship should be based on well-defined physical principles that can actually reproduce the observed coupling rather than modeling it using phenomenological assumptions. Here we are fortunate to have detailed and insightful experimental observations of the rotary–chemical behavior and torque generation for the F_1_-ATPase system, whose reproduction provides a powerful challenge for structure-based simulation approaches. In an effort to establish the origin of the rotary–chemical coupling and to simulate the experimental observations, it is important to clarify significant misunderstandings that have arisen. In particular, it is important to clarify that the experimentally observed torque and related observations cannot be reproduced without a proper coupling of the chemical and the conformational landscapes. Trying to force the γ stalk to rotate between different states of the enzyme can only reflect the applied TMD force used to generate the rotation (e.g. Pu & Karplus, 2008), rather than elucidate any inherent property of the system (stronger forces would simply lead to stronger response and faster rotation). The torque can be obtained from a structure-based simplified landscape of the entire system by several approaches, such as the one adopted in this study; calculating the PMF along the rotational degree of freedom by free-energy perturbation method on the rotary–chemical surface.

Our approach of combining the chemical energy profile with the CG conformational surface to give the rotary–chemical surface provided the unique ability to explore the possible role of having the catalytic dwell at different positions of the rotational angle. Our study indicates that despite nature's elegant design of an intriguing structure/energy relationship that results in having the chemical step at the 80° rotational angle, the position of the catalytic dwell does not seem to be essential for the torque generation process.

It would be worthwhile to note that the implications of the importance of dynamical effects in the chemical step (Watanabe *et al*. 2012) are interesting, but problematic at the conceptual level. This point has been demonstrated carefully in our work (Kamerlin & Warshel, 2010) where it was shown that the dynamical proposal (when formulated correctly) cannot account for any significant catalytic effect since it implies transfer of the conformational energy to the chemical coordinate and such energy is dissipated long before we have a productive fluctuation that leads to the chemical event. In other words, as shown in the only study that accurately simulated this effect (Pisliakov *et al*. 2009), the internal friction inside the protein completely dissipates the memory of the conformational motion in the millisecond time needed to wait for a productive chemical fluctuation. Interestingly, regardless of the conceptual basis, our findings are consistent with the findings of Watanabe *et al*. (2012) that concluded that the torque is influenced by the binding and release of the ligands and not so much by the catalytic event.

An interesting attempt to explore the conformational/chemistry landscape was presented in Watanabe *et al*. (2013) using phenomenological two-dimensional rotational/chemical surfaces. Although such an approach is insightful, it does not provide a unique connection to the underlying structure function correlation of the enzyme. That is, the chemical coordinate should reflect the free-energy changes along the α/β subunits conformational changes, as well as, the ATP hydrolysis of the specific bound ATP (see Fig. 2 of the present work and Fig. 3 of Mukherjee & Warshel, 2011). Unless the conformational landscape is known through structure based calculations (for e.g. the CG approach used in Mukherjee & Warshel, 2011), it is very hard to separate the effect of the chemical reaction from the conformational effect.

As stated in the methods section, we have studied the issue of coupling between the ATP hydrolysis and the conformation of the α/β subunits using the EVB approach in Strajbl *et al*. (2003) that also generated the catalytic free-energy surface for different ATPase configurations. The same issue was also explored by Dittrich *et al*. (2003) using a QM/MM energy minimization study that, however, did not involve proper sampling (see discussion in Kamerlin *et al*. 2013). The conformational dependence of the catalytic step has been explored recently by Beke-Somfai *et al*. (2013), who used QM/MM calculations and phenomenological description to model the process. It was concluded that the activation barrier is reduced significantly upon T to D change of the active site.

Considering the potential insights that can be gained from all atom explicit simulations (Warshel, 2014), it is quite tempting to assume that an understanding of molecular motor functionality can likewise be provided with all atom explicit models. Thus, it may seem reasonable to believe that the current reported all atom simulation approaches (Czub & Grubmuller, 2014; Nam *et al*. 2014; Okazaki & Hummer, 2013; Pu & Karplus, 2008) will be sufficient to provide a quantitative understanding of the underlying principles that can lead to unidirectional rotation in biologically relevant timescales generating the correct torque and can also reproduce the substep features along the rotary–chemical pathway of F_1_-ATPase. Here one must ask oneself, what is the actual information being provided by current atomistic simulation studies with explicit models and what is really needed in order to understand the rotary–chemical coupling with the currently available computational resources. It is needless to say that atomistic studies of local structural rearrangements in catalytic sites obtained from relatively smaller time scale simulation trajectories can benefit the understanding of the chemical, ligand binding and release processes greatly (for e.g. see Czub & Grubmuller, 2014; Nam *et al*. 2014; Okazaki & Hummer, 2013; Strajbl *et al*. 2003). However, the situation is very different when one tries to explore the molecular basis of the torque generation, unidirectionality and substep behavior of the enzyme occurring at much larger time scales. To realize the possible problems with explicit models it may be useful to consider a recent work (Nam *et al*. 2014) that claim to provide insight into the torque generation and rotary–chemical coupling in F_1_-ATPase. This work tried to explore the motion between the two X-ray structures close to the catalytic dwell and ATP waiting states (effectively near the 80° and 120° states shown in our study (Mukherjee & Warshel, 2011)), using an explicit all atom model and TMD simulations with huge external forces. The TMD simulations were done for systems with different P_i_ occupations in the β_E_ subunit, from the neighborhood of the catalytic dwell structure towards the ATP waiting structure. It was found that the system without P_i_ reached the ATP waiting state, while the one with P_i_ did not do so. Although this finding was presented as an advance in understanding the rotary–chemical coupling in the enzyme, it does not provide the needed information. That is, the difficulties of pushing one state to the other, by applying strong TMD forces within a very short time, does not tell us about the least free-energy path and functional free-energy barriers for the large-scale conformational changes. Similarly, such an approach does not provide a way to evaluate the torque generated during the actual biological process. In this respect, note that our previous CG study (Mukherjee & Warshel, 2011) has already reproduced the key features along the rotary–chemical surface before it has been suggested by Nam *et al*. (2014), namely, relatively small barrier for the 80° rotation (i.e. the ATP waiting state to the catalytic dwell state) followed by higher barriers for the 40° rotation (i.e. the catalytic dwell state to the next ATP waiting state), where the 40° rotation is tightly coupled to the α/β conformational changes. The key difference is that, in spite of providing interesting structural details of the key states involved along the rotary–chemical process, it is unlikely that even few TMD trajectories would capture the relevant free energies that can be used to generate a unified understanding of the F_1_-ATPase functionality.

The above discussion should serve as an illustration of the difficulties in using explicit models in trying to learn about the molecular origin of complex biological systems. Obviously, it is very tempting to assign observed functional behavior of the system to specific structural elements and to picoseconds-nanoseconds range dynamical features. However, a theoretically well-founded structure-based approach should attempt to reveal the mechanism of the complex biological system through construction of the relevant functional free-energy landscapes.

One of the most dominant proposals from some of the theoretical and experimental studies is that the torque in F_1_-ATPase is generated due to vdW or steric interactions (for e.g. see (Chiwata *et al*. 2014; Martin *et al*. 2014; Nam *et al*. 2014; Pu & Karplus, 2008). Unfortunately, such an implication based on the TMD simulations of F_1_-ATPase rotation is problematic (Nam *et al*. 2014; Pu & Karplus, 2008) as no proper calculation has yet established that the rotation is solely due to vdW interactions. In other words, implications that the studies of (Nam *et al*. 2014; Pu & Karplus, 2008) support the vdW idea overlooks the fact that these studies simply induced the rotation by TMD between the initial to final X-ray structures using large external force without evaluating the free-energy landscape and hence cannot correctly estimate the contribution of the vdW forces to such a landscape. The problem with the vdW idea is highlighted when one considers the experimental observations that the inherent F_1_-ATPase rotary property is still retained when one deletes major portion of the γ subunit embedded within the α/β subunit cavity (Chiwata *et al*. 2014). Moreover, one can only arrive at such conclusions after considering all aspects of the problem and after studying other relevant proposals, such as the role of electrostatics with valid theoretical models capable of doing so. Here it is important to realize that our studies actually produced for the first time a reasonable functional landscape for the rotary–chemical cycle (without forcing the system to rotate) and has obtained such a landscape through proper modeling of the long-range electrostatic forces of the system (Mukherjee & Warshel, 2011, 2015), while omitting the vdW forces (whose contributions to the landscape was found to be minimal). Furthermore, the basic paradigm of electrostatics-driven F_1_-ATPase has been able to reproduce the behavior of several γ-truncated systems studied experimentally and have also provided a detailed knowledge of important and redundant areas of the γ actually involved in the rotary–chemical coupling and the substep feature of the enzyme (Mukherjee & Warshel, 2015). In our view, our CG free-energy landscape studies has provided a major support for the electrostatics-driven mechanism, while one has yet to find any quantitative description of the proposed vdW driven mechanism of such processes.

Recent works has considered Brownian ratchet mechanism (Astumian, 2001; Frank & Gonzalez, 2010) as the underlying theoretical framework for many biological motors. This concept seems to stress the fact that ratcheted random thermal motions guide the action of molecular motors, but one still needs to elucidate the nature of the free-energy landscape that drives the ratcheting action. Once the free-energy surface is determined, the motor functionality and the corresponding vectorial processes are almost deterministic, since the individual rate constants follow the Boltzmann-based transition state theory. In fact, attributing functional features to fluctuations between several thermally accessible states (Frank & Gonzalez, 2010) (which might look to some as elements of a Brownian ratchet) is not so relevant to the question of how the motor operates. Having high chemical and conformational barriers is not fully compatible with the standard Brownian ratchet model and requires one to use a discrete chemical kinetics description (Kolomeisky & Fisher, 2007). Thus it is not useful to say that Brownian motions drive the motor (there are always thermal motions in all molecular systems), what counts are the rate limiting high barriers and the overall downhill free-energy profile, which is not related to or generated by the stochastic Brownian motions.

Overall, this study implies the fact that any special preference of the chemical event occurring at a specific rotational angle is not required for the efficient torque generation of F_1_-ATPase. The experimental finding of other ATPase systems where the catalytic dwell is at some other position apart from 80° (Furuike *et al*. 2011) can be used as an evidence that the specific position of the stall might not be crucial for efficient energy conversion and torque generation. However, one must note that the chemical energy is absolutely needed as the driving force for the torque generation, but its actual position of coupling to the rotational coordinate might not be important and might just be a result of the structural arrangement of the multi-subunit enzyme.

## References

[R1] Abrahams JP, Leslie AGW, Lutter R, Walker JE (1994). Structure at 2.8 Å resolution of F1-ATPase from bovine heart mitochondria. Nature.

[R2] Adachi K, Oiwa K, Nishizaka T, Furuike S, Noji H, Itoh H, Yoshida M, Kinosita K (2007). Coupling of rotation and catalysis in F(1)-ATPase revealed by single-molecule imaging and manipulation. Cell.

[R3] Arai HC, Yukawa A, Iwatate RJ, Kamiya M, Watanbe R, Urano Y, Noji H (2014). Torque generation mechanism of F-1-ATPase upon NTP binding. Biophysical Journal.

[R4] Astumian RD (2001). Making molecules into motors. Scientific American.

[R5] Beke-Somfai T, Lincoln P, Norden B (2013). Rate of hydrolysis in ATP synthase is fine-tuned by alpha-subunit motif controlling active site conformation. Proceedings of National Academy of Sciences of the United States of America.

[R6] Boyer PD (1993). The binding change mechanism for ATP synthase – some probabilities and possibilities. Biochimica et Biophysica Acta.

[R7] Boyer PD (1997). The ATP synthase – a splendid molecular machine. Annual Reviews of Biochemistry.

[R8] Chiwata R, Kohori A, Kawakami T, Shiroguchi K, Furuike S, Adachi K, Sutoh K, Yoshida M, Kinosita K (2014). None of the rotor residues of F-1-ATPase are essential for torque generation. Biophysical Journal.

[R9] Czub J, Grubmuller H (2014). Rotation triggers nucleotide-independent conformational transition of the empty beta subunit of F(1)-ATPase. Journal of the American Chemical Society.

[R10] Dittrich M, Hayashi S, Schulten K (2003). On the mechanism of ATP hydrolysis in F1-ATPase. Biophysical Journal.

[R11] Dryga A, Chakrabarty S, Vicatos S, Warshel A (2011). Coarse grained model for exploring voltage dependent ion channels. Biochimica et Biophysica Acta.

[R12] Fersht A (1999). Structure and Mechanism in Protein Science. A Guide to Enzyme Catalysis and Protein Folding.

[R13] Frank J, Gonzalez RL (2010). Structure and dynamics of a processive Brownian motor: the translating ribosome. Annual Reviews of Biochemistry.

[R14] Furuike S, Nakano M, Adachi K, Noji H, Kinosita K, Yokoyama K (2011). Resolving stepping rotation in *Thermus thermophilus* H(+)-ATPase/synthase with an essentially drag-free probe. Nature Communications.

[R15] Junge W, Sielaff H, Engelbrecht S (2009). Torque generation and elastic power transmission in the rotary F(O)F(1)-ATPase. Nature.

[R16] Kamerlin SC, Sharma PK, Prasad RB, Warshel A (2013). Why nature really chose phosphate. Quarterly Reviews of Biophysics.

[R17] Kamerlin SCL, Warshel A (2010). At the dawn of the 21st century: is dynamics the missing link for understanding enzyme catalysis?. Proteins-Structure Function and Bioinformatics.

[R18] Kamerlin SCL, Warshel A (2011). The empirical valence bond model: theory and applications. Wiley Interdisciplinary Reviews – Computational Molecular Science.

[R19] Kato M, Warshel A (2005). Through the channel and around the channel: validating and comparing microscopic approaches for the evaluation of free energy profiles for ion penetration through ion channels. Journal of Physical Chemistry B.

[R20] Kolomeisky AB, Fisher ME (2007). Molecular motors: a the orist's perspective. Annual of Reviews Physical Chemistry.

[R21] Martin JL, Ishmukhametov R, Hornung T, Ahmad Z, Frasch WD (2014). Anatomy of F-1-ATPase powered rotation. Proceedings of National Academy of Sciences of the United States of America.

[R22] Messer BM, Roca M, Chu ZT, Vicatos S, Kilshtain AV, Warshel A (2010). Multiscale simulations of protein landscapes: using coarse-grained models as reference potentials to full explicit models. Proteins.

[R23] Mukherjee S, Warshel A (2011). Electrostatic origin of the mechanochemical rotary mechanism and the catalytic dwell of F1-ATPase. Proceedings of National Academy of Sciences of the United States of America.

[R24] Mukherjee S, Warshel A (2015). Dissecting the role of the γ-subunit in the rotary–chemical coupling and torque generation in F_1_-ATPase. Proceedings of National Academy of Sciences of the United States of America.

[R25] Nam K, Pu J, Karplus M (2014). Trapping the ATP binding state leads to a detailed understanding of the F1-ATPase mechanism. Proceedings of National Academy of Sciences of the United States of America.

[R26] Noji H, Yasuda R, Yoshida M, Kinosita K (1997). Direct observation of the rotation of F1-ATPase. Nature.

[R27] Okazaki K, Hummer G (2013). Phosphate release coupled to rotary motion of F1-ATPase. Proceedings of National Academy of Sciences of the United States of America.

[R28] Okuno D, Fujisawa R, Iino R, Hirono-Hara Y, Imamura H, Noji H (2008). Correlation between the conformational states of F1-ATPase as determined from its crystal structure and single-molecule rotation. Proceedings of National Academy of Sciences of the United States of America.

[R29] Panke O, Cherepanov DA, Gumbiowski K, Engelbrecht S, Junge W (2001). Viscoelastic dynamics of actin filaments coupled to rotary F-ATPase: angular torque profile of the enzyme. Biophysical Journal.

[R30] Pisliakov AV, CAO J, Kamerlin SC, Warshel A (2009). Enzyme millisecond conformational dynamics do not catalyze the chemical step. Proceedings of National Academy of Sciences of the United States of America.

[R31] Pu J, Karplus M (2008). How subunit coupling produces the gamma-subunit rotary motion in F1-ATPase. Proceedings of National Academy of Sciences of the United States of America.

[R32] Sakaki N, Shimo-Kon R, Adachi K, Itoh H, Furuike S, Muneyuki E, Yoshida M, Kinosita K (2005). One rotary mechanism for F1-ATPase over ATP concentrations from milli-molar down to nanomolar. Biophysical Journal.

[R33] Shimo-Kon R, Muneyuki E, Sakai H, Adachi K, Yoshida M, Kinosita K (2010). Chemo-mechanical coupling in F(1)-ATPase revealed by catalytic site occupancy during catalysis. Biophysical Journal.

[R34] Strajbl M, Shurki A, Warshel A (2003). Converting conformational changes to electrostatic energy in molecular motors: the energetics of ATP synthase. Proceedings of National Academy of Sciences of the United States of America.

[R35] Uchihashi T, Iino R, Ando T, Noji H (2011). High-speed atomic force microscopy reveals rotary catalysis of rotorless F(1)-ATPase. Science.

[R36] Vicatos S, Rychkova A, Mukherjee S, Warshel A (2014). An effective coarse-grained model for biological simulations: recent refinements and validations. Proteins.

[R37] Wang H, Oster G (1998). Energy transduction in the F1 motor of ATP synthase. Nature.

[R38] Warshel A (2014). Multiscale modeling of biological functions: from enzymes to molecular machines (Nobel Lecture). Angewandte Chemie International Edition in English.

[R39] Watanabe R (2012). Mechanical modulation of catalytic power on F1-ATPase. Nature Chemical Biology.

[R40] Watanabe R, Hayashi K, Ueno H, Noji H (2013). Catalysis-enhancement via rotary fluctuation of F1-ATPase. Biophysical Journal.

[R41] Weber J, Senior AE (1997). Catalytic mechanism of F1-ATPase (Review). Biochimica et Biophysica Acta.

